# Efficacy of Music Therapy in Reducing Anxiety Among Postsurgical Cancer Patients: An Open-Label Randomized Controlled Trial

**DOI:** 10.7759/cureus.83392

**Published:** 2025-05-03

**Authors:** Rajeev Ranjan, Yash Jaiswal, Sulagna Mallik, Nidhi Joseph Varghese, Jagjit Kumar Pandey, Pankaj Kumar, Ratnadeep Biswas, Vishnu S Ojha

**Affiliations:** 1 Psychiatry, All India Institute of Medical Sciences, Patna, Patna, IND; 2 Surgical Oncology, All India Institute of Medical Sciences, Patna, Patna, IND

**Keywords:** anxiety, cancer, depression, music therapy, pain, stress

## Abstract

Background: Surgery is one of the key therapeutic modalities for cancers such as breast and colon cancer. Researchers claim that poorly managed anxiety slows recovery in postsurgical cancer patients and requires proactive management, including mind-body therapies like music therapy, which may further help reduce opioid consumption. In this study, we aimed to assess the efficacy of music therapy in reducing anxiety, depression, stress, and pain among postsurgical cancer patients over two weeks.

Methods: This was a randomized, open-label parallel design trial with a sample size of 44 participants divided into two groups: an intervention group A (n = 22) that received music therapy along with the standard treatment regimen, and a control group B (n = 22) that only received the standard treatment regimen. We used curated music tracks. We assessed anxiety and other psychological symptoms (depression and stress) using the Depression, Anxiety, and Stress Scale-21 (DASS-21) and analyzed pain symptoms using the visual analog scale (VAS) at baseline and after two weeks.

Results: After the two-week period of music therapy as an adjuvant, we observed a significant reduction in anxiety scores (1.73; 95% confidence interval (CI): 0.0817 to 3.37; p = 0.040; Cohen’s d = 1.35) in the intervention group compared to the control group. For depression (1.09; 95% CI: -0.26 to 2.81; p = 0.207; Cohen’s d = 1.04) and pain (0.273; 95% CI: -1.23 to 1.77; p = 0.716; Cohen’s d = 0.42), scores in the intervention group also showed reductions compared to the control group, although these were statistically not significant.

Conclusion: Our findings suggest a significant improvement in anxiety scores with two weeks of adjuvant music therapy. Thus, music can be used as a non-invasive adjunctive therapeutic to manage anxiety symptoms in postoperative cancer patients. We recommend further studies on music therapy as an adjuvant treatment for patients with cancer undergoing palliative care.

## Introduction

Cancer is a chronic disease that remains one of the leading causes of death. Researchers estimate that in recent years, cancer has caused over 9.5 million deaths worldwide, which amounts to approximately one in six of all deaths. Based on available data, experts forecast that cancer cases and cancer-related deaths may rise to 21.4 million and 13.2 million, respectively, by 2030. If we examine the available statistics, the most common tumors in male patients are prostate, lung, and colon/rectum cancers, while in female patients, cancers affecting the breasts, lungs, and colon/rectum are fairly common. According to longitudinal data pertaining to cancer incidence, primarily from high-income countries, researchers observe a significant decrease in cancer-related deaths. We can credit these decreases in cancer cases and cancer-related deaths to improved detection and treatment, as well as advances in the field of surgery [1].

Surgery is quite common in cases of solid tumors localized in a specific area. Some common procedures include radical mastectomy for breast cancer, colostomy for colon cancer, and cholecystectomy for cholangiocarcinoma [2]. It is commonly found that patients undergoing surgical procedures often experience pain, stress, anxiety, and depressive symptoms post-operation [3,4]. Surgery is performed in cancer cases for diagnostic or treatment purposes [2]. According to experts, poorly managed postoperative pain and anxiety slow patient recovery. It is essential to proactively manage pain, anxiety, and stress to reduce anxiolytic and opioid consumption and improve overall quality of patient care after surgery [5].

The emotional appeal of music is essentially universal and has measurable effects on emotional experience [6]. Several studies point to a deep-seated relationship between music-evoked emotions and the specific parts of the nervous system that it affects. It is evident that music-evoked emotions significantly impact emotion-processing areas of the brain, namely the amygdala, hippocampus, anterior cingulate cortex, nucleus accumbens, and orbitofrontal cortex [7]. Given that the evidence indicates music alters neuronal activity, further studies are essential to explore the vast therapeutic capabilities of music.

Music can be an effective, adjunctive, non-pharmacological approach to managing pain, stress, and anxiety in postoperative cancer patients. The author of a recent meta-analysis found that music improves the body’s immune system function and reduces the stress hormone cortisol [8]. Additionally, researchers have found that music has clinical utility in various settings, from operating rooms to family clinics [9]. Current literature, mostly from overseas, shows a significant impact of music on reducing pain and anxiety in cancer patients [5,6]. Authors of one study found that active engagement in music, as well as tailor-made music interventions, reduce acute pain and associated anxiety symptoms, helping patients reconnect with healthy parts of themselves [10]. We require more indigenous studies to evaluate similar effects in Indian patients [11]. India’s sociocultural and economic factors significantly influence attitudes toward non-pharmacological interventions in health settings [10]. There is a dearth of studies demonstrating the efficacy of music as adjunct therapy in treating anxiety and mood among cancer patients post-surgery. Therefore, we aimed to evaluate the efficacy of music therapy as an adjuvant therapy for anxiety, along with depressive, stress, and pain symptoms in postsurgical cancer patients in India.

Study objectives

The primary objective of the study was to compare the effects of music therapy and standard of care against standard of care alone on anxiety symptom severity in postsurgical cancer patients over a two-week period, using the Depression, Anxiety, and Stress Scale-21 (DASS-21). Secondary objectives included comparing the effects of music therapy and standard of care against standard of care alone on depression, stress, and pain symptoms severity in postsurgical cancer patients, over the same two-week period, using DASS-21 and visual analog scale (VAS).

## Materials and methods

We conducted the study at the Department of Psychiatry in collaboration with the Department of Surgical Oncology at All India Institute of Medical Sciences, Patna, Bihar, India, after obtaining approval from the Institute Ethics Committee (AIIMS/Pat/IEC/2022/909) following the Indian Council of Medical Research’s National Ethical Guidelines for Biomedical and Health Research involving human participants. We registered the study with the Clinical Trials Registry - India (2023/08/056019).

Study population and eligibility

We included patients aged 18-65 years who had been operated on by the surgical oncology team and were stable in postoperative clinical care at the surgical oncology ward after receiving their informed consent for the therapeutic intervention and participation in the study. We excluded individuals with significant hearing and other sensory impairments or serious medical conditions hindering communication, as well as uncooperative patients. Individuals with significant cognitive impairments, diagnosed comorbid psychiatric conditions, and neurological disorders, including those caused by brain tumors and metastasis, were also excluded through clinical interviews and thorough clinical examinations.

Study design, procedure, and setting

This study was a prospective, randomized, open-label parallel design trial conducted in a single tertiary care center in India. We obtained written informed consent from all participants after explaining the diagnosis, nature, purpose, and benefits of the study. We collected detailed medical histories and performed clinical evaluations using a semi-structured proforma at baseline. We randomized participants by mixed block randomization into two treatment groups with an allocation ratio of 1:1. We assigned codes to the sequence of random numbers and concealed allocation using a method involving serially numbered opaque sealed envelopes.

**Figure 1:**
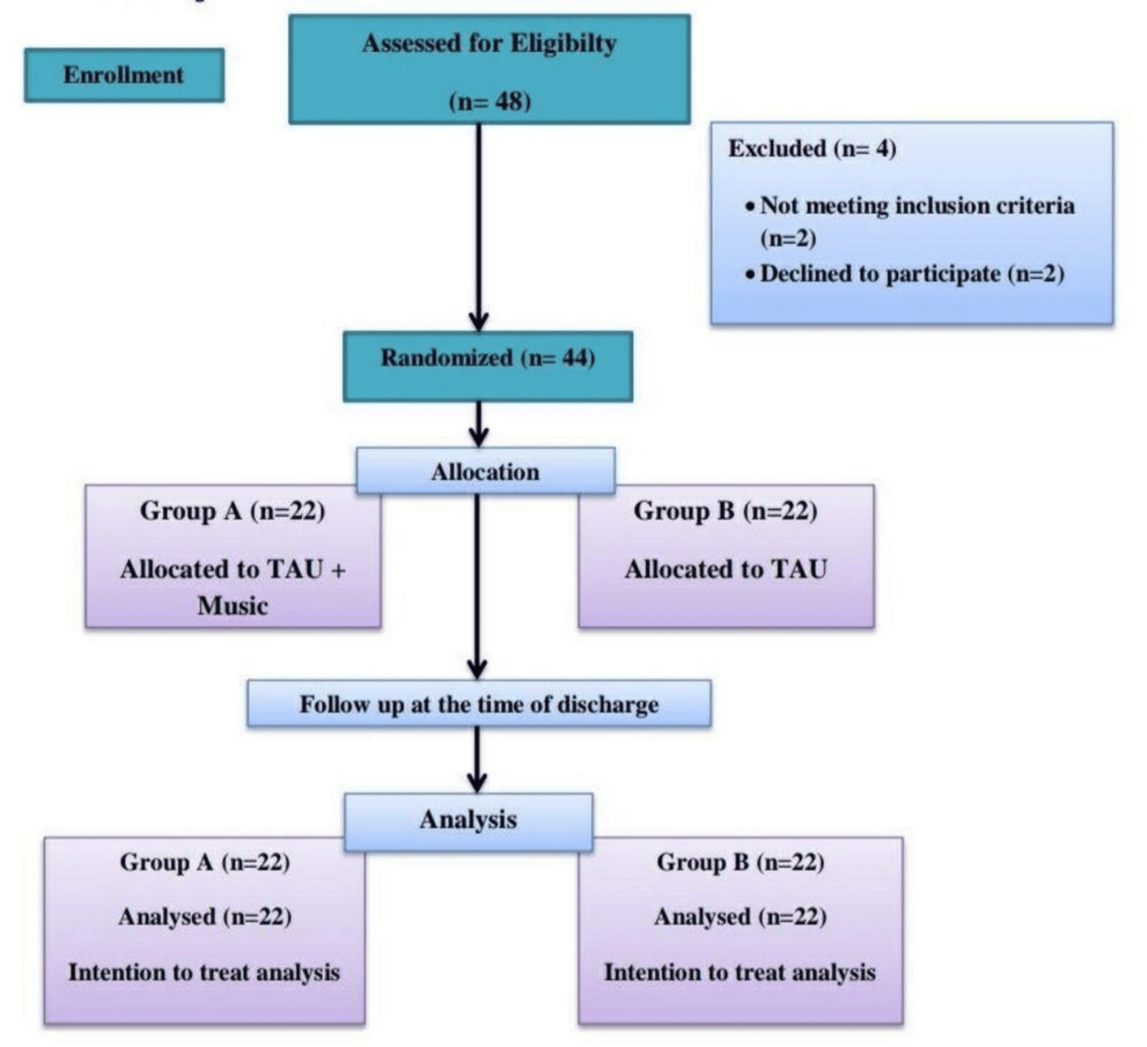
CONSORT diagram showing the flow of participants through each stage of the randomized trial CONSORT: Consolidated Standards of Reporting Trials; TAU: Treatment as usual

After randomization, we divided patients into two treatment groups. Group A patients received music therapy along with standard of care, while Group B patients received only standard of care. Both groups received brief psychoeducation to begin. In both groups, the standard of care/treatment as usual (TAU) included antibiotics, analgesics, and nutritional support. Subsequently, we conducted baseline assessments using the DASS-21 for anxiety, depression, and stress, as well as the VAS for pain. Music therapy was based on the preferences of participants selected in Group A. We first allowed Group A patients to listen to a music track in a group session just before the personalized intervention to address their activation levels (baseline levels of anxiety, stress, pain, and mood states) and familiarize them with the music, followed by personalized sessions, administered with the help of headphones, each lasting 30 minutes. We carried out these sessions daily over a period of two weeks for patients who were undergoing postsurgical cancer care. Clinical assessments were conducted once at baseline and again after two weeks using the DASS-21 and VAS to evaluate the reduction in symptom severity (Figure 1).

Intervention (music therapy)

We selected the music for the prescribed music program based on a literature review and music characterization system, sourcing it from available data banks of music files on various platforms (Spotify, YouTube, etc.). The tracks used in this study included Indian classical tunes, which researchers have shown to be effective in managing postoperative anxiety and pain relief [12]. Other scholars have demonstrated that such tunes reduce depression, anxiety, and stress symptoms among elderly adults [13]. The study also incorporated Indian instrumental music, which has proven beneficial in alleviating depression, stress, anxiety, and pain symptoms [14]. The tailored music tracks featured not only Indian classical ragas but also instrumental music that has shown promising effects on depression and anxiety [15]. Additionally, the collection included a number of Western songs, which, as demonstrated in a study conducted by MindLab, are quite helpful in reducing depression, stress, and anxiety symptoms [16,17]. We mixed these to create 30-40 minute prerecorded music sessions tailored to patient preferences for administration over two weeks, without any lyrics. Each session began by attuning the patient according to their baseline activation level (based on the theory of entrainment) [18]. Based on activation levels, we played this music for the first 5-7 minutes in a group and then individually. The remaining 23-30 minutes comprised a personalized session designed for relaxation, with the music progressively transitioning to a slower tempo, intensity, and movement through the session, eliciting simultaneous physiological and emotional responses within the patient. Those on the no music therapy protocol did not receive any music therapy; however, we offered music therapy upon request in subsequent follow-up appointments after the study duration.

Outcome measures

The primary outcome measure was the change in anxiety scores on DASS-21 from baseline to two weeks. The secondary outcome measures included: (a) change in depression and stress scores on DASS-21 from baseline to two weeks and (b) change in pain scores on VAS from baseline to two weeks.

Tools used for the measurement of study outcomes 

*DASS-21* 

This tool primarily assesses three negative emotional states: depression, anxiety, and stress. It consists of 21 items, with seven items dedicated to each of anxiety, depression, and stress, measured on a four-point rating scale (0-3) [19]. The total score ranges from 0 to 63. The reliability of the DASS-21 is evidenced by its excellent Cronbach's alpha values of 0.81, 0.89, and 0.78 for the stress, anxiety, and depression subscales, respectively. Its discriminative, concurrent, convergent, and internal consistency have also been evaluated as excellent [20]. The DASS-21 has been validated as a reliable tool for populations with cancer, with reliability coefficients (McDonald’s omega) ranging from 0.84 to 0.95 and demonstrating moderate to strong correlations with well-being indicators and stress measures [21]. 

VAS

The VAS is a commonly used outcome measure for pain, represented as a 100 mm horizontal line scored from 1 to 10. Pain intensity is indicated by a point on the line between “no pain at all” and “worst pain imaginable.” Its simplicity, reliability, validity, and ratio scale properties make the VAS an optimal tool for describing pain severity and intensity. The VAS has shown good test-retest reliability with an intra-class correlation (ICC = 0.81, p < 0.001), and its correlation with known groups (r = 0.99, p < 0.001) and convergent validity (r = 0.78, p < 0.001) is also good, collectively demonstrating solid construct validity in cancer patients with stable symptoms [22]. 

Sample size determination

We estimated the sample size using an online sample size calculator (https://statulator.com). Taking a 5% level of significance and 80% power, we calculated a mean difference for postoperative anxiety between the two groups as 4.28 with a standard deviation (SD) of 0.53 [9]. The calculated sample size for detecting the difference in mean postoperative anxiety scores between the two groups was determined to be 44, with 22 participants in each group. 

Statistical analyses

We used descriptive statistics, including frequency, percentages, mean, and SD. We compared means and proportions of continuous and categorical variables between groups using the chi-squared test, Fisher’s exact test, or independent samples t-test for the demographic profile. We assessed the normality of the primary and secondary outcome datasets at two weeks using the Shapiro-Wilk test. Changes in DASS-21 and VAS scores were compared within groups using paired t-tests and between groups using independent t-tests. All statistical analyses were performed using an intention to treat (ITT) approach with JAMOVI version 2.3.24 (for MacOS) (Jamovi, Sydney, Australia), considering a significance level of p < 0.05.

## Results

Recruitment process, participant demographics, and baseline characteristics

We enrolled a total of 48 postoperative cancer patients in the study, of whom two patients did not meet the inclusion criteria, and two patients declined to participate. Since the major endpoint was two weeks following the patients' admission while they were still in the hospital, there were no dropouts during follow-up in either group. A total of 22 patients were present in each group after randomization, and we analyzed them with ITT (Figure 1) as the primary analysis. We did not consider per-protocol analysis as a secondary measure because there were no dropouts in our study.

There was no significant difference in demographic parameters between the two treatment groups (Table 1) at baseline. The mean age of cancer patients requiring surgery in both groups was 49.9 ± 13.8 years and 49.1 ± 17.1 years (mean ± SD), respectively, which was almost comparable. Most participants in both groups were married (86.36% vs 77.27%), Hindu by religion (91% vs 91%), and from joint families (68.18% vs 72.72%). A larger number of participants were male (54.5% vs 54.5%), literate (54.5% vs 54.5%), and unemployed (54.5% vs 63.64%) (Table 1).

**Table 1:** Baseline demographic data and clinical characteristics (n = 44) MT: Music therapy; TAU: Treatment as usual; SD: Standard deviation @: Unpaired t test; #: Fisher’s exact test; *: Chi-squared test

Characteristics		MT + TAU (n = 22)	TAU (n = 22)	t-value^@^/ chi-squared value*/ Fisher's exact^#^	p-value
Age, mean (SD)		49.9 (13.8)	49.1 (17.1)	0.165^@^	0.870
Gender	Male	12 (54.55%)	12 (54.55%)	0.228*	0.228
	Female	10 (45.45%)	10 (45.45%)		
Education	Illiterate	10 (45.45%)	10 (45.45%)	0.248*	0.248
	Literate	12 (54.55%)	12 (54.55%)		
Marital status	Unmarried	3 (13.6%)	5 (22.7%)	0.611*	0.434
	Married	19 (86.36%)	17 (77.27%)		
Occupational status	Unemployed	12 (54.54%)	14 (63.64%)	1.47*	0.226
	Employed	10 (45.45%)	8 (36.36%)		
Religion	Hindu	20 (91%)	20 (91%)	0.226^#^	0.635
	Muslim	2 (9%)	2 (9%)		
Family type	Nuclear	7 (31.81%)	6 (27.27%)	0.109*	0.741
	Joint	15 (68.18%)	16 (72.72%)		
Locality	Rural	14 (63.64%)	11 (50%)	0.834*	0.361
	Urban	8 (36.36%)	11 (50%)		
Types of cancer	Gastrointestinal cancer	5 (22.77%)	7 (31.81%)	0.275*	0.785
	Head and neck cancer	4 (18.19%)	4 (18.19%)		
	Breast cancer	6 (27.23%)	4 (18.19%)		
	Others	7 (31.81%)	7 (31.81%)		

The distribution of cancer types requiring surgery in both groups was also nearly matched. The majority of cases were gastrointestinal carcinoma, including instances such as rectal carcinoma, colon cancer, and cholangiocarcinoma (22.77% vs 31.81%); head and neck cancer, which included carcinoma of the tongue and carcinoma of the gingiva-buccal sulcus (27.23% vs 18.19%); and carcinoma of the breast (31.81% vs 31.81%). Other cases included testicular cancer, ovarian cancer, and carcinoma of the forearm (18.19% vs 18.19%) (Table 1).

Test for normality for the primary and secondary outcomes

The Shapiro-Wilk test was applied to check the normality of the dataset for the primary and secondary outcomes at the end of two weeks. As depicted in Table 2, in both treatment groups, the data were normally distributed since the p-value was greater than 0.05 in both groups (Table 2); thus, we applied appropriate statistical tests (paired t-test/independent t-test for within/between groups) for the normally distributed data.

**Table 2:** Shapiro-Wilk test for normality of primary and secondary outcome measures (anxiety, depression, stress, and DASS-21 subscores; pain score on VAS) at the end of two weeks MT: Music therapy; TAU: Treatment as usual; DASS-21: Depression, Anxiety and Stress Scale-21; VAS: Visual analog scale

Outcomes	Intervention groups	n	Mean	p-value
Primary outcome				
Anxiety	MT + TAU	22	5.32	0.408
	TAU	22	4.14	0.305
Secondary outcomes				
Depression	MT + TAU	22	3.50	0.102
	TAU	22	2.41	0.809
Pain	MT + TAU	22	4.68	0.563
	TAU	22	4.27	0.111
Stress	MT + TAU	22	5.14	0.452
	TAU	22	5.95	0.503

Change in anxiety score (primary outcome measure)

We found that the anxiety score, as measured by DASS-21, was at the lower end after two weeks compared to baseline, with statistically significant changes (p < 0.001) in both groups (music vs no music). In the intervention group (music therapy), the difference was significant between baseline and the end of two weeks, with a mean difference of 5.86 (95% confidence interval (CI): 4.56 to 7.16, p < 0.001). In the control group (no music therapy, only standard of care), the difference was significant between baseline and follow-up, yielding a mean difference of 4.27 (95% CI: 3.23 to 5.32, p < 0.001). The music therapy group performed significantly better than the control group, as the mean difference in anxiety scores between the intervention (music) and control groups (no music, only standard of care) at the end of two weeks, per ITT analysis, was 1.73 (95% CI: 0.0817 to 3.37; Cohen’s d = 1.35) and was statistically significant (p = 0.040) (Table 3).

**Table 3:** Change in anxiety, stress, depression (DASS-21 scores) and pain (VAS scores) in/across study groups over a period of two weeks (n = 44) as per ITT analysis MT: Music therapy; TAU: Treatment as usual; DASS-21: Depression, Anxiety and Stress Scale-21; VAS: Visual analog scale; ITT: Intention to treat; SD: Standard deviation; CI: Confidence interval $: Paired t-test; @: Independent t-test

Variables	Group A (MT + TAU) (n = 22)				Group B (TAU) (n = 22)				Difference between Group A and Group B		Cohen’s d
	Baseline mean (SD)	Follow-up mean (SD)	Mean difference (95% CI)	p-value $	Baseline mean (SD)	Follow-up mean (SD)	Mean difference (95% CI)	p-value $	Mean difference (95% CI)	p-value @	
Anxiety	7.09 (4.15)	1.23 (1.00)	5.86 (4.56–7.16)	<0.001	5.32 (5.00)	1.05 (0.94)	4.27 (3.23–5.32)	<0.001	1.73 (0.081–3.37)	0.040	1.35
Pain	5.59 (2.12)	1.45 (2.61)	4.14 (3.12–5.15)	<0.001	5.55 (2.69)	0.955 (0.65)	4.59 (3.49–5.69)	<0.001	0.273 (-1.23–1.77)	0.716	0.42
Stress	6.68 (4.02)	1.55 (2.32)	5.14 (3.91–6.36)	<0.001	7.00 (4.60)	1.05 (2.28)	5.95 (4.37–7.14)	<0.001	-0.818 (-2.76–1.12)	0.400	-0.57
Depression	5.59 (5.26)	2.05 (2.80)	3.55 (2.31–4.97)	<0.001	3.50 (3.69)	1.18 (2.11)	2.32 (1.22–3.42)	<0.001	1.09 (-0.627–2.81)	0.207	1.04

Change in depression score (secondary outcome measure)

We also found that the depression score was lower at the end of two weeks compared to baseline, with statistically significant results (p < 0.001) in both the intervention and control groups. The music therapy group performed numerically better than the control group, as the mean difference in depression scores between the intervention and control groups at two weeks was 1.09 (95% CI: -0.26 to 2.81; Cohen’s d = 1.04), but this was statistically not significant (p = 0.207) (Table 3).

Change in stress score (secondary outcome measure)

We found that the stress score was lower at the end of two weeks compared to baseline, and it was statistically significant (p < 0.001) in both the intervention and control groups. The music therapy group did not perform numerically better than the control group, as the mean difference in stress scores between the groups at two weeks was -0.818 (95% CI: -2.76 to 1.12; Cohen’s d = -0.57), and it was statistically not significant (p = 0.400) (Table 3).

Change in pain score (secondary outcome measure)

We found that the pain score was lower at the end of two weeks compared to baseline, with statistically significant results (p < 0.001) in both intervention and control groups. The music therapy group performed numerically better than the control group, with the mean difference in pain scores between the groups at two weeks being 0.273 (95% CI: -1.23 to 1.77; Cohen’s d = 0.42), but this difference was statistically not significant (p = 0.716) (Table 3).

## Discussion

Our study aimed to determine the effect of a non-invasive, accessible, soothing therapeutic modality - music - on psychological outcomes (depression, anxiety, and stress) and physical outcomes (pain) among postoperative cancer patients. We are likely the first researchers to use music therapy as an adjuvant treatment in postsurgical cancer patients in this subcontinent. We assessed parameters of depression, anxiety, and stress using DASS-21 and the pain parameter using VAS, which are standardized, simple, and easy tools to administer. After two weeks of music therapy as an adjuvant, we found that the intervention group's anxiety scores were significantly lower than those of the control group. Meanwhile, the intervention group's scores for depression and pain were lower than those of the control group at the end of two weeks, but they were not statistically significant.

Listening to music elicits a specific emotional effect that has a deep-seated stimulative relationship with specific regions of the brain (amygdala, hippocampus, anterior cingulate cortex, nucleus accumbens, and orbitofrontal cortex) responsible for emotion processing [7]. Music also affects the release of corticotropin-releasing hormone from the pituitary gland, which eventually slows the release of cortisol, thereby inducing a feeling of relaxation and preventing stress-induced responses in the body [16]. In our study, we focused on the effects of music therapy over two weeks on postoperative cancer patients who had been admitted to a surgical oncology ward. Although numerous studies have assessed the effects of music therapy on postoperative patients [10,18,23], our study aimed to analyze the effects of music therapy on postoperative cancer patients in an Indian tertiary healthcare center, making it unique. There is a dearth of clinical trials and studies involving the use of tailor-made music tracks on postoperative cancer patients in an Indian healthcare setting. In this study, we made an advancement using standardized music based on an extensive literature review [10,11]. 

Our study’s primary objective included investigating the effect of music therapy on anxiety symptoms in postoperative cancer patients. Results indicated that the reduction in anxiety scores was significant compared to the control group and from baseline in our intervention group. This suggests that music therapy is effective in reducing anxiety among postoperative cancer patients. These findings align with those of other studies that showed a significant reduction in anxiety scores in the intervention group compared to the control group in postoperative patients [10,24]. Authors of one of these studies explained that the reduction in anxiety slows tumor growth in cancer patients by lowering the pro-inflammatory cytokine levels, suggesting that music therapy may help alleviate anxiety [24]. 

The secondary objectives of our study were to observe the effect of music therapy on pain and depressive symptom scores of postoperative cancer patients, which we assessed using DASS-21 and VAS. We noted that the reduction in pain and depression scores was significant compared to baseline in our intervention group, and the music therapy group performed better than the control group, though not significantly. This may be attributed to a higher number of participants with gastrointestinal carcinoma (who underwent procedures such as gastrectomy) in the control group, who were suffering from chronic pain and somatization symptoms of depression. Researchers have also found similar results in studies conducted on patients with chronic non-malignant tumors, which showed no significant reduction in pain and depressive symptom scores in the intervention group compared to the control group [25,26]. Another secondary objective of our study was to examine the effect of music therapy on stress scores of postoperative cancer patients. Results indicated a significant reduction in stress scores from baseline in our intervention group, but no significant difference in stress scores between groups. These findings resonate with another study on postoperative patients, which found no significant reduction in stress hormone cortisol levels in the intervention group receiving music therapy compared to the control group [27]. The negative findings of our secondary study outcomes may be due to our sample population not being adequately powered for the secondary outcomes. 

Our study has some limitations. First, no preoperative assessments were conducted, and a two-week duration of assessment may limit the long-term efficacy of music therapy on anxiety scores in postoperative cancer care. Hospital discharge could have alleviated such symptoms. A longer follow-up duration would have provided deeper insights into the effects of music therapy on anxiety symptoms among postoperative cancer patients. Second, a study focused on the effects of music therapy on patients with a specific type of cancer (more homogenous) would have strengthened the study. Third, sample size estimation for the secondary outcomes would have generated more evidence for music therapy as an adjunct therapy in cancer care. Fourth, we could not define a superiority margin for our study since we could not locate any robust guidelines for identifying a superiority margin for therapy in pain, palliative, and cancer care. A superiority margin could have enhanced the validity of our results. Fifth, some items of the DASS-21 may be affected by cancer, surgery, and medication use; however, we took utmost care while collecting our data. Sixth, we could not assess the sustainability of treatment effects after the two-week period. Lastly, an open-label design poses a risk for multiple biases; however, we utilized an open-label design for this trial due to the difficulty in blinding investigators and participants because of the types of interventions used in both arms. Nonetheless, our study was the first of its kind to assess the role of music therapy in cancer patients in an Indian population. It suggests that music therapy can serve as a relatively safe adjunct to medications to improve the quality of life in postsurgical cancer patients.

## Conclusions

To conclude, our study findings suggest a significant improvement in anxiety symptoms with two weeks of adjuvant music therapy. Music, as an adjuvant therapy, can be utilized as a non-invasive, holistic, patient-centered, non-pharmacological, complementary intervention to manage anxiety symptoms in postoperative cancer patients. Music therapy can enhance emotional well-being and support recovery. Although the clinical implications are promising, further research is necessary to refine treatment protocols and identify better tracks with promising therapeutic value, which can enhance quality of life and emotional resilience during the challenging postoperative period for cancer patients. 
